# Effects of Reheating Methods on Rheological and Textural Characteristics of Rice Starch with Different Gelatinization Degrees

**DOI:** 10.3390/foods11213314

**Published:** 2022-10-22

**Authors:** Jiani Jiang, Jiangtao Li, Wenfang Han, Qiqi Yang, Qiongxiang Liu, Huaxi Xiao, Qinlu Lin, Yong Fang

**Affiliations:** 1 National Engineering Research Center of Rice and Byproduct Deep Processing, School of Food Science and Technology, Central South University of Forestry and Technology, Changsha 410004, China; 2 College of Food Science and Engineering, Nanjing University of Finance and Economics/Collaborative Innovation Center for Modern Grain Circulation and Safety, Nanjing 210023, China

**Keywords:** pregelatinized starch, gelatinization degree, microwave, rheology, texture, heating mode

## Abstract

Pregelatinized starch (PGS) is often used to improve the processing quality of foodstuffs, but little attention has been paid to the effects of different reheating methods and degree of starch gelatinization (DSG) on their rheological and textural properties. In this study, pregelatinized rice starches (RS) with gelatinization degrees ranging from 58% to 100% were prepared via different Rapid Visco Analyser (RVA) heating procedures and reheated in various methods, including high-power microwave (HM), low-power microwave (LM), and water bath. The rheological behavior and textural properties were explored, and the results demonstrated that the consistency, gel strength, hardness, and springiness of PGS in all tested samples decreased significantly after reheating. The storage modulus (G’) of PGS increased dramatically while the thermal stability decreased. Interestingly, the reheating methods possessed various effects on the starch of different DSG.

## 1. Introduction

Convenience is a major trend in the food industry [1]. The impact of pneumonia caused by the novel coronavirus (2019-nCoV) has increased the need for consumers to “eat at home”, but the current cooking process is complicated and time-consuming [2]. With the development of the ‘cold chain’, the popularity of microwave ovens and other heating equipment, ready meals (frozen, chilled, and shelf-stable convenience foods), which are pretreated and require only simple heating by consumers, have rapidly captured a large market [3,4]. Notably, starchy ready-to-heat foods such as instant rice, instant vermicelli, frozen dumplings, and semifinished steamed buns are popular and appealing at this particular time. Therefore, the processing and application characteristics of starchy ready-to-heat food products are worth exploring.

Gelatinization, which is one of the most important properties of starch, refers to the process when starch granules are heated to a certain temperature in water, the volume of the starch granules increases, and the hydrogen bonds between the molecules are broken due to expansion and the ordered structures are destroyed, forming viscous paste-like solution [5]. Compared with native starch, pregelatinized starch (PGS), a class of physically modified starch, could significantly decrease viscosities of the starch paste, weaken gel structures, and affect rheological properties as well as retrogradation characteristics [6,7,8,9]. Therefore, the PGS is often applied to improve food quality and edibleness, such as ameliorating the taste and texture of breads [10], increasing the tensile strength of noodles [11], improving the texture and nutritional properties of dumplings [12], and the physical and physicochemical properties of instant rice and its eating quality [13]. Recent research has demonstrated that the properties of PGS are strongly associated with the source, amylose content, water content, and degree of starch gelatinization (DSG) of PGS [9,11,14,15]. Notably, DSG is the crucial quality index of PGS and one of the primary factors affecting the structure and physicochemical properties of starch [5]. Xu et al. [16] studied physicochemical and structural characterization of potato starch with different degrees of gelatinization, and the results showed that the starch granule structure disappeared more significantly with increasing DSG. Moreover, the starch gel formed after reheating has higher viscoelasticity, but the molecular mechanism of the corresponding gelation behavior was still unclear. Reheating is a predominant part of prepared food processing, and microwave processing—an efficient heating method—has attracted great attention in academic research and industry for its application in reheating [17,18]. In the traditional water bath heating method, heat is transferred from the external high-temperature food area or the bottom of the heated container to the low-temperature area in the food center. In contrast, the polar molecules vibrate at a high frequency and collide with other molecules in the food during the microwave heating process, converting kinetic energy into heat, and the heat transfer in microwave heating is in the food surface and interior simultaneously, thus shortening the time of heating [19,20]. It has been reported that the morphology and crystallinity of starch granules are more severely damaged by microwaves than by conventional heat treatment, probably due to the strong friction and collision generated by electromagnetic waves [21,22]. The rheological properties of starch can predict and explain flow property, deformation, and texture changes of various starch-based foods. As far as we know, few studies have focused on the comprehensive impact of reheating method and difference in DSG on the rheological and textural properties of PGS. We systematically studied the different effects of microwave reheating and water bath reheating on the rheological and textural properties of starches with different DSG in anticipation of provide a theoretical basis for the further processing and application of PGS. It is important to guide the reheating process and maintain the product quality of starchy ready-to-heat foods, especially frozen semifinished starchy food.

## 2. Materials and Methods

### 2.1. Materials

Dongtingchun soft rice was purchased from Hunan Dongtingchun Rice Industry Co., Ltd. (Changde, China). All other chemicals were of analytical grade and obtained from commercial sources.

### 2.2. Sample Preparation

#### 2.2.1. Preparation of Rice Starch [23]

Rice starch was prepared using alkaline extraction method. The rice was first washed 2–3 times with water, then soaked in the volume of 0.3% (W/V) NaOH 3 times and replaced with new NaOH every 8 h for 24 h. After wet milling by colloidal mill, the rice starch slurry was centrifuged at 3500 g for 10 min, while the supernatant and yellow precipitate were discarded, and only the white starch layer remained. The white product was then soaked in 0.3% NaOH and stirred for 15 min. After being kept static, the supernatant was discarded and NaOH solution was added, and the procedure was repeated many times within 24 h. Later, the above solution was adjusted to pH = 7 with HCl (0.1 mol/L) and then the white centrifugal precipitate was repeatedly washed utilizing distilled water until the supernatant was free of precipitate (using 1% AgNO_3_ to test). The end-products were dried in an electric blast oven (DHG-9070A, Yiheng Scientific Instrument Co., Shanghai, China) and ground for further use.

#### 2.2.2. Preparation of Rice Starch with Different Gelatinization Degrees

A specimen of 3 g starch was added to 25 mL of distilled water and then different heating procedures of RVA (Table 1) were used to prepare starch with gelatinization degrees of 58%, 80%, and 100%, respectively [24]. The gelatinization degree was determined according to the enzymatic method described by Liu et al. [25], and the samples were successively named as low-gelatinized starch (LGS), medium-gelatinized starch (MGS), and high-gelatinized starch (HGS) in that order. During this preparation process, the paddle rotating speed was first maintained at 960 rpm for 10 s, and then kept constant at 160 rpm throughout the entire process thereafter. Finally, each sample was quickly cooled, then freeze-dried using a vacuum freeze dryer (F05-4T, sim international, Beijing, China), and ground into powders for further analyses.

### 2.3. Preparation of Reheat Rice Starch by Conventional Water Bath and Microwave Heating

#### 2.3.1. Microwave Reheating

After standing at 4 °C for 24 h, the pastes (prepared according to the heating procedure in Table 1) were subjected to microwave reheating treatment using a microwave oven (G90F23CN3XLVN-R6 (TM), Galanz Ltd., Guangdong, China). The center of 30 g LGS, MGS, or HGS paste were inserted into an infrared thermometer and reheated with high-power microwave (HM) (3.0 W/g sample) for 2 min to enable center temperature of the sample to reach 85 °C. They were denoted as LGS-HM, MGS-HM, and HGS-HM, respectively. Similarly, the low-power microwave (LM) (1.8 W/g sample) was used to heat the samples with different DSG for 3 min with a central temperature of 85 °C, which were denoted as LGS-LM, MGS-LM and HGS-LM, respectively. After reheating, the samples were freeze-dried and ground for further use.

#### 2.3.2. Water Bath Reheating

Similarly, 30 g starch pastes of LGS, MGS, or HGS were stored at 4 °C for 24 h. The centers of these pastes were inserted into an infrared thermometer and then reheated in a boiling-water bath for 30 min to reach a center temperature of 85 °C. After reheating, these samples were freeze-dried and ground and denoted as LGSW, MGSW, and HGSW, respectively.

### 2.4. Scanning Electron Microscopy (SEM)

The observation of microscopic morphology was executed as previously described [9]. In briefly, the microstructures of the freeze-dried and gold-sprayed starch samples were observed using a scanning electron microscope (SU8010, Hitachi, Tokyo, Japan) with an acceleration voltage of 10 KV.

### 2.5. Fourier Transform Infrared (FTIR) Spectroscopy

The short-range ordered molecular structure was tested by the approach of Zhang et al. [26]. In briefly, sample preparation was carried out using the KBr pellet method and FTIR spectrometer was recorded using an FTIR spectrometer (IRTracer-100, Shimadzu, Kyoto, Japan). The spectra were collected in the range 4000–400 cm^−1^ with a resolution of 4 cm^−1^, and a total of 64 scans was performed for all samples. Each sample was scanned three times and the spectrum was analyzed by Omnic software. 

### 2.6. Water-Binding Capacity (WBC) and Water Solubility Index (WSI)

WBC and WSI were determined by referring to the method described by Xu et al. [16] and Obadi et al. [11] with slight revisions. A total of 1 g of starch (dry wt. basis) was dispersed in 50 mL of distilled water and agitated for 30 min at 50 °C, 60 °C, 70 °C, 80 °C, and 90 °C, separately. Then, the above samples were cooled rapidly to room temperature with cold water and centrifuged (4000 rpm) for 30 min. A total of 10 mL of the supernatants obtained after centrifugation were dried at 105 °C for 4 h to determine the amount of dissolved starch. WBC and WSI were calculated according to the following formula:

WBC (%) = [(wet weight of starch − dry weight of original starch)/dry weight of original starch] × 100(1)

WSI (%) = (weight of dry supernatant/dry weight of original starch) × 100(2)

### 2.7. Static Rheology

A rheometer (DHR-2, TA Instruments Ltd., Crawley, UK) with the rheometer plate (40 mm diameter) and a 1 mm gap was used to measure the rheological properties of the samples. The starch solutions (6%, W/V) were heated in a boiling-water bath for 0.5 h, and after cooling to 25 °C they were transferred to the rheometer measurement platform. The change of shear stress was measured at a measuring temperature of 25 °C. The data acquisition and recording were performed automatically by computer. The static starch rheology data were modeled by using the power law Equation (3).

τ = k*γn(3)

where τ is the shear stress (Pa), k is the consistency index (Pa s^n^), γ˙ is the shear rate (s^−1^), and n is the flow behavior index (dimensionless).

### 2.8. Dynamic Rheology

After being solubilized, starch solution was maintained at 25 °C under magnetic stirring for 40 min, and then rheological test at this temperature was performed. A constant frequency of 1 Hz was used in the test, and the strain amplitude for the frequency sweep measurements was chosen to be 1%, which was in the linear viscoelastic region for all samples. For constant dynamic rheology properties of the samples, the temperature was carried out with heating (25–95 °C) and cooling gradients (95–25 °C) at a rate of 3 °C/min. Small-amplitude oscillatory shear flows with frequencies from 0.01 to 10 Hz were used to measure storage modulus (G′) and loss modulus (G″).

### 2.9. Gel Texture

The gel texture properties were measured via a Texture Analyser (TA-XT-PIUS, Stable Micro Systems, Godalming, UK) following the previous description with slight modifications [12]. A total of 3 g starch sample was weighed and placed in an RVA box. After gelatinization with RVA, it was poured into a Petri dish with a height of 10 mm, a diameter of 60 mm, and smooth surface. The starch paste used in the RVA test was placed in a 4 °C refrigerator for 24 h, and then cut into 1 cm^3^ pieces of the same size. The gel strength of the samples was measured with a P/0.5 cylinder probe. The procedure was to move down and compress the gel to 10 mm of the original sample height at a rate of 2 mm/s. After contacting the sample with a trigger force of 5 g, the test speed was set to 1.0 mm/s and the post-test speed was set to 0.5 mm/s. The strength of the gel structure is the product of the maximum force (g) and the maximum distance (mm) required to compress the sample. The hardness, cohesiveness, gumminess, and springiness of the gels were conducted using a P/36R cylinder probe with a two-cycle program at a compression ratio of 50%, a trigger force of 5 g, and a test speed of 1 mm/s. Each measurement was repeated 5 times. 

### 2.10. Blue Value (BV)

The iodine-binding properties of starch are characterized by the iodine blue value, which was executed as previously described [27]. A total of 10 mg (dry basis) of the sample was dissolved in 20 mL distilled water and shaken in a boiling-water bath until the sample was completely dissolved to prepare a 2 mg/mL starch solution. After cooling them to room temperature, 1 mL of each was added to 1 mL of iodine reagent (2 mg/mL I_2_, 20 mg/mL KI) and the volume was fixed to 100 mL with distilled water. Then, the absorbance was measured at 680 nm after being placed in the dark for 15 min. 

### 2.11. Statistical Analysis

The data were expressed as mean ± standard deviation. One-way ANOVA and multiple comparison tests were performed using IBM SPSS software version 20.0 (Chicago, Illinois, USa). *p* < 0.05 was considered statistically significant.

## 3. Results and Discussion

### 3.1. Microscopic Morphology

**Figure 1:**
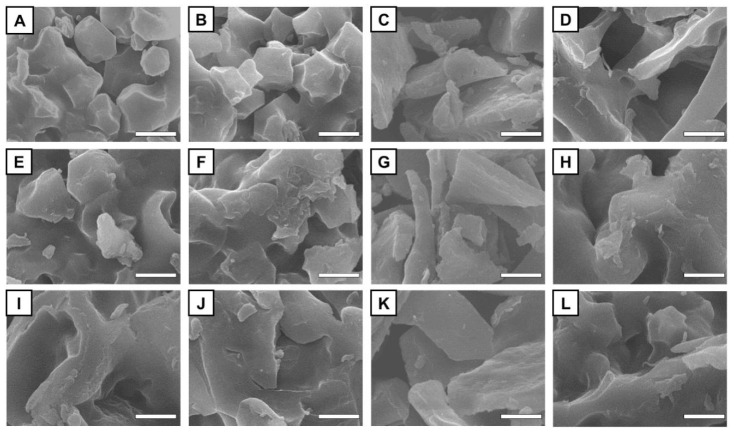
SEM images of PGSs reheated by different methods. ((**A**): LGS; (**B**): LGS-HM; (**C**): LSG-LM; (**D**): LGSW; (**E**): MGS; (**F**): MGS-HM; (**G**): MGS-LM; (**H**): MGSW; (**I**): HGS; (**J**): HGS-HM; (**K**): HGS-LM; (**L**): HGSW). Scale bars = 5 μm.

To evaluate the effects of reheating methods on the microstructure of starches with different gelatinization degrees, the low-, medium- and high-gelatinized starch samples were reheated by microwave or water bath individually. Representative micrographs for starches with different gelatinization degrees before and after reheating are shown in Figure 1. Results showed that the structure of LGS (Figure 1A) is irregular, smooth, and polyhedral. As the DSG increased, the starch granules began to fracture, collapsed into flakes or lumps, and gradually lost their typical polyhedral shape. This phenomenon was mainly caused by the pregelatinized processing resulted starch granules irreversible swelling and microcrystalline melting [16]. In consistent with the results of Li et al., the starch granules in all tested PGSs were destructed by reheating, and the destruction extent is correlated with the gelatinization degree [28]. The effect of reheating mode on the microstructure was more pronounced in LGS. The particle surfaces of LGS-HM (Figure 1B) and LGS-LM (Figure 1C) became rougher or even broken. Moreover, the structural damage severity of LGS was positively correlated with microwave power, while the polyhedral structure was still maintained. The surface structure of LGS particles was more complete in microwave-reheated samples than that in water bath-reheated ones; this could be explained by the short duration of the microwave reheating process could not completely dissolve the amylose in LGS. The MGS-HM (Figure 1F) and HGS-HM (Figure 1J) particles show the highest degree of surface fragmentation in the medium- and high-gelatinized starch samples. This result can be caused by the rapid heat generation and the penetration of heat into starch granules through microwave-energized molecular friction, resulting in greater damage to starch granules than water bath reheating [21]. The damage severity of starch granule structure increased as the power of the microwave treatment increased, as the collision and friction between molecules enhanced with the microwave treatment power. 

### 3.2. Short-Range Ordered Molecular Structure

**Figure 2:**
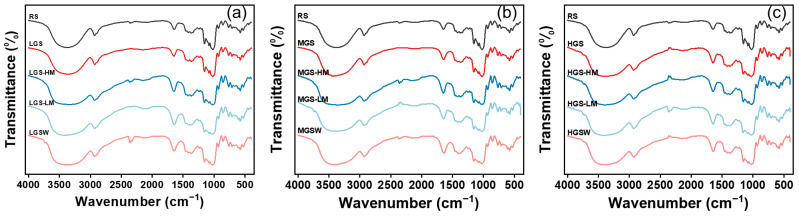
FTIR spectra of LGS (**a**), MGS (**b**) and HGS (**c**) reheated by different methods.

The FTIR tests were performed on PGSs with different gelatinization degrees, and the results are presented in Figure 2 and Table 2. All starch samples showed similar absorbance peaks in the FTIR spectra, indicating that the pregelatinization and reheating had no apparent effect on the chemical composition of the rice starch and did not result in new chemical groups in the starch molecules. The bands at 995 cm^−1^, 1047 cm^−1^, and 1022 cm^−1^ were related to the hydrogen bonds formed between hydroxyl groups of starch macromolecules, ordered structure, and amorphous structure in starch, respectively [29]. The ratios of absorbance at 1047/1022 cm^−1^ and 995/1022 cm^−1^ are commonly used to measure changes in the short-range ordered structure of starch.

As for nonreheated samples, the values of both R1047/1022 and R995/1022 ratio were similar in RS and LSG, respectively. Moreover, the values of these ratios increased as DSG increased. This result indicated that storage of PGSs at 4 °C could generate a higher short-range ordered structure. This may be due to the reorganization of starch chains and double-helix structures through intermolecular and intramolecular hydrogen bonding [30]. After reheating, R1047/1022 and R995/1022 ratios increased first and then kept constant as DSG increased in the tested samples. For both MGS and HGS, all samples with or without reheating showed similar ratios in R1047/1022 and R995/1022, indicating that reheating did not significantly affect the short-range ordered structure of PGS, which is consistent with the findings of Xu et al. [9]. 

### 3.3. Static Rheology

**Figure 3:**
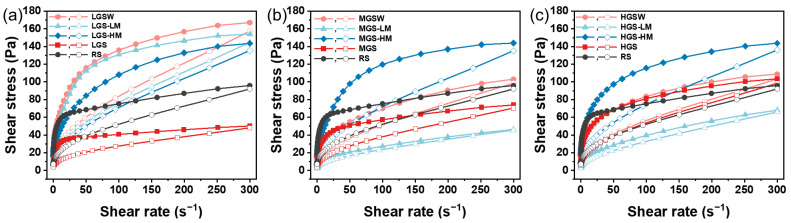
Static rheological curve of LGS (**a**), MGS (**b**), and HGS (**c**) reheated by different methods.

Rheological properties refer to the deformation and flow properties under the action of external force. The variation trends of shear stress following shear rate changes in PGS of different DSGs with/without reheating is shown in Figure 3. As starch is a non-Newtonian fluid, the corresponding shear stress increased as the shear rate rose in all samples.

To further analyze the static rheological properties of starch paste, the data were modeled by a power law equation. As shown in Table 3, the R^2^ values of all samples were between 0.969 and 0.996, indicating that Equation (3) can nicely fit the static rheological data of starches with different DSG. In addition, all starch samples exhibited a lower flow behavior index (n < 1), which was attributed to shear strain breaking the molecular network in the starch paste [31]. During the upward (0.1~300/s) shearing, all PGSs exhibited a higher consistency index (K) and lower flow behavior index (n), and their consistency index decreased while the flow behavior index increased after reheat treatment. In addition, the microwave-treated samples experienced greater change than water bath-treated samples. These results demonstrate that the reheating methods had significant effects on the rheological behavior of PGSs with different DSG. Meanwhile, the starch paste became more elastic and less viscous under microwave reheating, which was consistent with previous research results [9,32]. During the downward (300~0.1/s) shearing, apart from HGSW, the consistency indexes of reheated PGSs were lower than RS. However, the flow behavior index did not show regular changes, which may be related to the thixotropy of the samples. The areas of the hysteresis loop of the PGSs were reduced after reheating, indicating a reduction in the thixotropy and an improvement in the stability and resilience of the starch paste. Notably, this property is beneficial to starch processing and production. 

### 3.4. Dynamic Rheology

**Figure 4:**
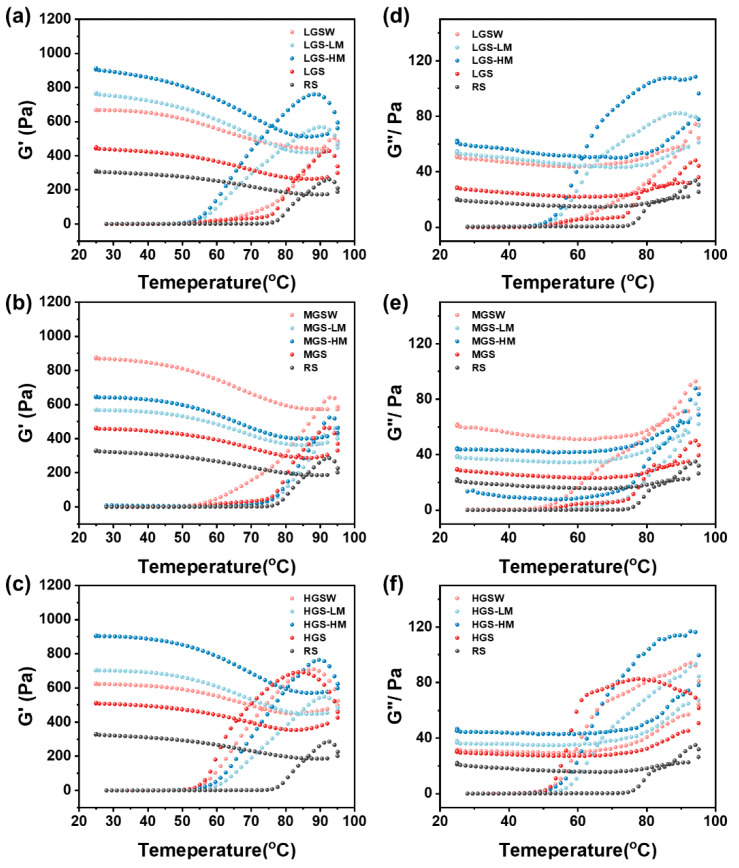
Development of G′ (**a**–**c**) and G″ (**d**–**f**) of starch pastes reheated by different methods.

G′ and G″ reflect the elasticity and viscosity of starch, respectively [33]. Figure 4 shows the temperature dependence of G′ and G″ for each group of samples. All starch samples showed similar fluid behavior, and the change of modulus was strongly dependent on temperature. In addition, G′ and G″ enhanced with the increase in DSG at the same temperature, but the changes in G″ were not as significant as G′. These results are in agreement with the results of static rheology studies, which demonstrated that a more stable three-dimensional network structure can be formed with the increase in DSG. 

During the heating process (25 °C to 95 °C), the increasing amplitude of G′ showed its highest level when the temperature was below the gelatinization temperature. A major reason for this phenomenon may be that the starch did not gelatinize during the initial heating stage, so the system was unstable and the viscoelasticity varied considerably. However, as the starch granules continued to break up with heating, amylose dissolved and interconnected to form a network structure, which greatly increased the elasticity of the system, while the viscosity increased slower. When the temperature was higher than the gelatinization temperature, the prolonged reheating time caused partial cleavage of the hydrogen bonds between the starch gel structures, thereby increasing the mobility of amylose and the viscosity of the starch system. During the cooling process, the changes of both G′ and G″ slowed down, which was probably because the starch molecules were hydrogen-bonded to the water molecules, and resulted in a stabilization of the gel viscoelasticity. In addition, MGS showed the highest peak G′ after water bath treatment rather than microwave reheating, which was different from LGS and HGS, suggesting that the reheating methods had different effects on the rheological behavior of PGSs with different DSG. These results can be explained by MGS having the highest amylose content and the amylose leaching during the reheating process enhancing the interaction with the granules.

### 3.5. Gel Texture

After gelatinization and retrogradation, starch molecules can form a dense three-dimensional gel network, the gel properties of which can be closely related to the processing characteristics of starchy foods and the quality of the products [34]. Thus, we measured the gel strength of PGSs after reheating treatment (Table 4) and evaluated the effects of different reheating methods on the texture characteristics of starch samples as well, and the results are shown in Table 5. 

The reheated PGS exhibited significant changes in the texture characteristics, including hardness, cohesiveness, springiness, and gumminess. The hardness and springiness dramatically reduced, which may be related to the regelatinization of starch during the reheating process. The reheating treatment led to an increase in the damages to the crystalline region of starch granules and a decrease in the stability of the gel structure. In addition, the gumminess of all PGSs increased after reheating. The gumminess increase was greater in microwave reheating, especially in the low-power microwave treatment group, in comparison with that in the traditional water bath reheating method. This may be due to the degradation of long-chain starch molecules to short chain starch by the nonthermal effect of microwaves. These short-chain starch molecules could swell and stretch more fully during gelatinization, resulting in an increase in the overall viscosity of the starch gel. Hence, the low-power microwave treatment group with a longer treatment time increased gumminess more significantly in comparison to high-power microwave processed samples. It is noteworthy that MGS always had a relatively larger gel strength after reheating compared to LGS and HGS, probably because LGS was incompletely gelatinized, while the molecular chain of HGS were broken during the reheating process and resulted in the destruction of the gel network structure.

### 3.6. Water-Binding Capacity (WBC) and Water Solubility Index (WSI)

**Figure 5:**
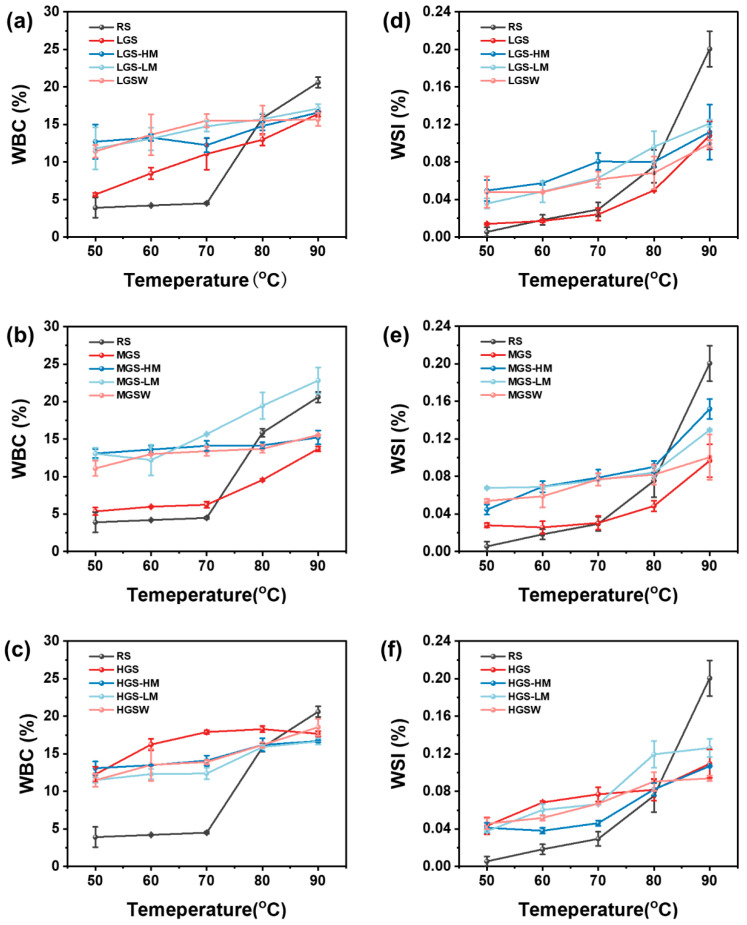
WBC (**a**–**c**) and WSI (**d**–**f**) of PGSs reheated by different methods.

The WBC and WSI of starch are important indicators to evaluate its physical properties, and could reflect the tightness of the internal structure of starch granules to a certain extent. The WBC and WSI of the RS and PGSs before and after reheating are presented in Figure 5. The results showed significant increases in the WBC and WSI values when the reheating temperature increased from 50 °C to 90 °C. All PGSs exhibited higher WBC and WSI than RS when the dissolving temperature ranged between 50 °C to 70 °C. Similarly, a previous study suggested that this observation may be related to the disruption of the granule structure and the gelatinization of the starch [16,35]. It was possible that the dissociation of the double-helix structure of starch during pregelatinization allowed free water molecules to penetrate easily into the interior of the starch molecules, thus facilitating the dissolution of the starch granules. This speculation can be further supported by the results shown in Figure 1. During water bath reheating, starch swelling was limited by the rearrangement of molecular chains and the formation of amylopectin side-chain clusters [36]. This may primarily explain the lower WBC of all PGSs at 80 °C and 90 °C compared with RS after water bath reheating.

### 3.7. Blue Value

**Figure 6:**
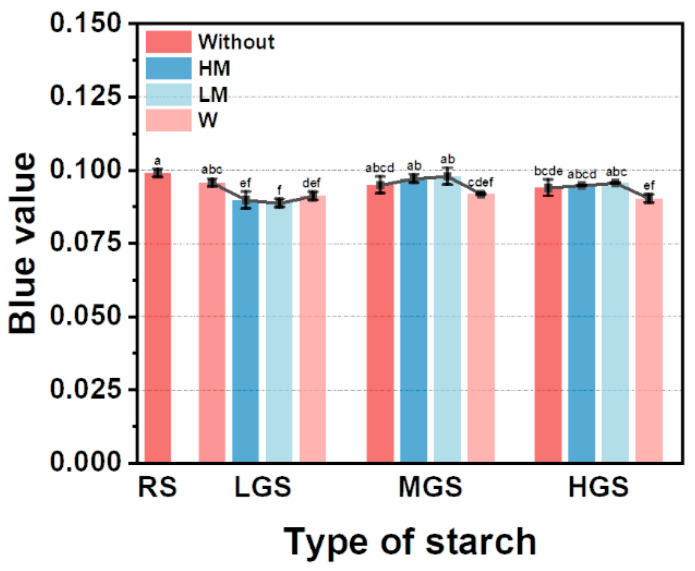
Blue value of PGSs reheated by different methods. Different letters indicate significant differences (*p* < 0.05).

Effects of reheating on the iodine blue values of PGSs of different DSG are shown in Figure 6. During the gelatinization process of starch granules, the long amylose molecules were broken into amylose with shorter chain length, which affected the binding of starch molecules to iodine. Thus, the BV values declined after water bath reheating in all tested samples, whereas the iodine blue value increased in the microwave-reheated samples. This may be due to the partial breaking of the amylopectin side chain, which increases the content of iodine-binding amylose molecules.

## 4. Conclusions

Reheating methods affect the rheological behavior, textural properties, and molecular structure of starches with different DSG differentially. Meanwhile, rice starch paste is a non-Newtonian fluid with shear-thinning behavior, and its rheological properties conform to the rheological law τ = k*γn. Starch paste with higher DSG tends to form a more stable three-dimensional network structure with higher gel strength. The variation amplitude of storage modulus increased significantly in PGSs with different DSG after reheating. In addition, due to a shorter microwave treatment time to reach the same temperature compared with the conventional water bath heating method, as well as the dual effect of microwave radiation and dielectric heating, the microwave-treated PGS has a rougher granule surface structure, poorer molecular structure stability, and higher blue values compared to samples reheated by water bath. Moreover, these starch pastes possess lower hardness and springiness, while exhibiting higher gumminess. MGS always had the relatively larger gel strength after reheating than RS, LGS, and HGS after reheating, and thus has great application potential in starch/food research and industrial areas. These results may be related to the interaction between amylose and starch granules, but the concrete mechanism and how our findings can guide the production of starchy ready-to-heat food as well as processing applications remains to be investigated by further experiments.

## Figures and Tables

**Table 1:** RVA gelatinization procedures.

LGS		MGS		HGS	
Time (hh:mm:ss)	Temperature (°C)	Time (hh:mm:ss)	Temperature (°C)	Time (hh:mm:ss)	Temperature (°C)
00:00:00–00:00:10	50	00:00:00–00:00:10	50	00:00:00–00:00:10	50
00:00:10–00:01:00	50	00:00:10–00:01:00	50	00:00:10–00:01:00	50
00:01:00–00:04:42	70	00:01:00–00:04:42	71	00:01:00–00:04:42	95
00:04:42–00:06:42	70	00:04:42–00:07:12	71	00:04:42–00:07:12	95
00:06:42–00:11:00	50	00:07:12–00:11:00	50	00:07:12–00:11:00	50
00:11:00–00:13:00	50	00:11:00–00:13:00	50	00:11:00–00:13:00	50
00:13:00	0	00:13:00	0	00:13:00	0

**Table 2:** Ratios of absorbance at 1047/1022 cm^−1^ and 995/1022 cm^−1^ of starch samples.

Type of Starch	R1047/1022	R995/1022
RS	0.80 ± 0.01 ^c^	0.75 ± 0.05 ^b^
LGS	0.83 ± 0.08 ^bc^	0.80 ± 0.06 ^ab^
LGS-HM	0.90 ± 0.01 ^ab^	0.87 ± 0.01 ^a^
LGS-LM	0.94 ± 0.04 ^a^	0.87 ± 0.03 ^a^
LGSW	0.90 ± 0.01 ^ab^	0.82 ± 0.08 ^ab^
MGS	0.84 ± 0.08 ^bc^	0.80 ± 0.01 ^ab^
MGS-HM	0.91 ± 0.04 ^ab^	0.88 ± 0.03 ^a^
MGS-LM	0.92 ± 0.01 ^ab^	0.86 ± 0.09 ^ab^
MGSW	0.92 ± 0.02 ^ab^	0.84 ± 0.01 ^ab^
HGS	0.92 ± 0.01 ^ab^	0.83 ± 0.01 ^ab^
HFS-HM	0.91 ± 0.02 ^ab^	0.84 ± 0.06 ^ab^
HFS-LM	0.92 ± 0.04 ^ab^	0.88 ± 0.04 ^ab^
HGSW	0.90 ± 0.04 ^ab^	0.81 ± 0.02 ^a^

Data are means ± SD. Different letters in the same column of data indicate significant differences (*p* < 0.05).

**Table 3:** The power law fitting parameters of starch pastes with different DSG reheated by different methods ^1^.

Type of Starch	Upward Curve			Downward Curve			
	K (Pa s^n^)	n	R^2^	K (Pa s^n^)	n	R^2^	Area of Hysteresis Loop
RS	10.94	0.33	0.973	9.44	0.32	0.969	1800.06
LGS	11.26	0.35	0.974	10.09	0.32	0.975	2051.93
LGS-HM	3.77	0.42	0.989	5.32	0.32	0.993	506.62
LGS-LM	3.79	0.42	0.990	5.32	0.32	0.991	622.15
LGSW	5.31	0.40	0.994	5.25	0.37	0.985	977.46
MGS	15.05	0.31	0.988	12.15	0.30	0.977	3604.88
MGS-HM	4.96	0.45	0.995	6.02	0.37	0.987	875.42
MGS-LM	5.15	0.44	0.996	6.03	0.37	0.992	890.41
MGSW	5.66	0.37	0.995	5.26	0.36	0.995	908.89
HGS	15.87	0.31	0.975	15.46	0.29	0.974	1114.09
HGS-HM	5.30	0.34	0.994	5.93	0.30	0.990	548.85
HGS-LM	7.42	0.35	0.995	7.90	0.30	0.992	563.62
HGSW	10.92	0.36	0.990	14.14	0.29	0.980	633.09

^1^ Where K is the consistency index (Pa s^n^), n is the flow behavior index (dimensionless), and R^2^ is the coefficient of determination.

**Table 4:** Gel strength of starch pastes with different DSG reheated by different methods.

Type of Starch	Without	HM	LM	W
HGS	753.73 ± 29.65 ^b^	475.84 ± 11.24 ^a^	482.82 ± 12.20 ^a^	490.57±22.49 ^a^
MGS	736.83 ± 26.88 ^a^	495.45 ± 26.28 ^a^	514.65 ± 16.34 ^a^	495.79±18.80 ^a^
LGS	710.95 ± 31.67 ^a^	400.57±10.27 ^b^	407.50 ± 14.32^b^	476.16±20.61 ^a^
RS	628.44 ± 23.34 ^ab^	-	-	-

Data are means ± SD. Different letters in the same column of data indicate significant differences (*p* < 0.05).

**Table 5:** Texture properties of starch pastes with different DSG reheated by different methods.

Type of Starch	Hardness (g)	Cohesiveness	Springiness (m)	Gumminess (g)
RS	38.58 ± 2.75 ^c^	0.46 ± 0.02 ^bc^	80.65 ± 1.49 ^ab^	17.96 ± 1.49 ^a^
LGS	52.14 ± 0.73 ^f^	0.43 ± 0.00 ^cd^	89.83 ± 3.54 ^d^	29.74 ± 0.18 ^d^
LGS-HM	24.30 ± 3.21 ^de^	0.41 ± 0.03 ^d^	74.43 ± 4.98 ^d^	21.26 ± 1.22 ^cd^
LGS-LM	24.46 ± 1.99 ^b^	0.59 ± 0.01 ^a^	74.90 ± 1.97 ^a^	25.81 ± 1.22 ^a^
LGSW	27.36 ± 2.08 ^ef^	0.45 ± 0.03 ^bcd^	75.07 ± 1.17 ^bcd^	18.04 ± 1.39 ^cd^
MGS	43.36 ± 3.86 ^b^	0.48 ± 0.01 ^b^	86.32 ± 1.31 ^a^	28.08 ± 3.31 ^a^
MGS-HM	34.11 ± 3.40 ^a^	0.41 ± 0.01 ^d^	72.74 ± 1.01 ^bc^	15.76 ± 1.56 ^c^
MGS-LM	36.47 ± 0.61 ^c^	0.60 ± 0.01 ^a^	78.48 ± 1.06 ^abc^	21.88 ± 0.87 ^a^
MGSW	38.08 ± 2.84 ^cd^	0.45 ± 0.01 ^bcd^	79.85 ± 6.96 ^cd^	12.01 ± 2.18 ^cd^
HGS	43.45 ± 2.49 ^b^	0.46 ± 0.00 ^bc^	81.62 ± 2.61 ^ab^	19.99 ± 1.16 ^bc^
HGS-HM	28.65 ± 3.66 ^d^	0.49 ± 0.01 ^b^	75.39 ± 1.23 ^bcd^	14.17 ± 0.18 ^cd^
HGS-LM	28.62 ± 1.05 ^d^	0.59 ± 0.01 ^a^	75.96 ± 2.14 ^a^	17.98 ± 2.37 ^ab^
HGSW	30.61 ± 0.43 ^d^	0.45 ± 0.01 ^bcd^	76.39 ± 1.26 ^bcd^	12.81 ± 0.32 ^cd^

Data are means ± SD. Different letters in the same column of data indicate significant differences (*p* < 0.05).

## Data Availability

The data presented in this study are available on request from the corresponding author. The data are not publicly available due to restrictions (for example, privacy of research participants).

## References

[1] Buckley M., Cowan C., McCarthy M. (2007). The Convenience Food Market in Great Britain: Convenience Food Lifestyle (CFL) Segments. Appetite.

[2] Piochi M., Buonocore F., Spampani F., Torri L. (2022). Impact of COVID-19 Lockdown on the Perception of Home Meals and Meal-Related Variables: A Large-Scale Study within the Italian Population during the Acute Phase of the Pandemic. Food Qual. Prefer..

[3] Wooldridge K., Riley M.D., Hendrie G.A. (2021). Growth of Ready Meals in Australian Supermarkets: Nutrient Composition, Price and Serving Size. Foods.

[4] Jackson P., Viehoff V. (2016). Reframing Convenience Food. Appetite.

[5] Zhao T., Zhang H., Chen F., Tong P., Cao W., Jiang Y. (2022). Study on Structural Changes of Starches with Different Amylose Content during Gelatinization Process. Starch Stärke.

[6] Kankate D., Panpalia S.G., Kumar K.J., Kennedy J.F. (2020). Studies to Predict the Effect of Pregelatinization on Excipient Property of Maize and Potato Starch Blends. Int. J. Biol. Macromol..

[7] Wang X., Wang H., Song J., Zhang Y., Zhang H. (2018). Understanding the Structural Characteristics, Pasting and Rheological Behaviours of Pregelatinised Cassava Starch. Int. J. Food Sci. Technol..

[8] Adedokun M.O., Itiola O.A. (2010). Material Properties and Compaction Characteristics of Natural and Pregelatinized Forms of Four Starches. Carbohydr. Polym..

[9] Xu F., Liu W., Zhang L., Danthine S., Liu Q., Wang F., Zhang H., Hu H., Blecker C. (2022). Retrogradation and Gelling Behaviours of Partially Gelatinised Potato Starch as Affected by the Degree of Pre-Gelatinisation. Int. J. Food Sci. Technol..

[10] Sugiura F., Ito S., Arai E. (2017). Effect of Pregelatinized Starch Paste on the Ease of Swallowing High-Moisture Content Bread. J. Food Eng..

[11] Obadi M., Chen Y., Qi Y., Liu S., Xu B. (2020). Effects of Different Pre-Gelatinized Starch on the Processing Quality of High Value-Added Tartary Buckwheat Noodles. Food Meas..

[12] Wang H., Xiao N., Wang X., Zhao X., Zhang H. (2019). Effect of Pregelatinized Starch on the Characteristics, Microstructures, and Quality Attributes of Glutinous Rice Flour and Dumplings. Food Chem..

[13] Prasert W., Suwannaporn P. (2009). Optimization of Instant Jasmine Rice Process and Its Physicochemical Properties. J. Food Eng..

[14] Zhao X., Xu X., Jin Y., Xu D., Zhang W., Wu F. (2021). Differences in Retrogradation Characteristics of Pregelatinized Rice Starch Prepared Using Different Water Content. Starch Stärke.

[15] Nakorn K.N., Tongdang T., Sirivongpaisal P. (2009). Crystallinity and Rheological Properties of Pregelatinized Rice Starches Differing in Amylose Content. Starch Stärke.

[16] Xu F., Zhang L., Liu W., Liu Q., Wang F., Zhang H., Hu H., Blecker C. (2021). Physicochemical and Structural Characterization of Potato Starch with Different Degrees of Gelatinization. Foods.

[17] Napp T.A., Gambhir A., Hills T.P., Florin N., Fennell P.S. (2014). A Review of the Technologies, Economics and Policy Instruments for Decarbonising Energy-Intensive Manufacturing Industries. Renew. Sustain. Energy Rev..

[18] Jiang H., Liu Z., Wang S. (2018). Microwave Processing: Effects and Impacts on Food Components. Crit. Rev. Food Sci. Nutr..

[19] Casasnovas J., Anantheswaran R.C. (2016). Dynamic Measurement of Starch Granule Swelling during Microwave Heating. Carbohydr. Polym..

[20] Li J., Han W., Xu J., Xiong S., Zhao S. (2014). Comparison of Morphological Changes and in Vitro Starch Digestibility of Rice Cooked by Microwave and Conductive Heating. Starch Stärke.

[21] Cui R., Yeon Yoo M.J., Zhu F. (2022). Comparison of Microwave and Conventional Heating on Physicochemical Properties and Phenolic Profiles of Purple Sweetpotato and Wheat Flours. Food Biosci..

[22] Bilbao-Sáinz C., Butler M., Weaver T., Bent J. (2007). Wheat Starch Gelatinization under Microwave Irradiation and Conduction Heating. Carbohydr. Polym..

[23] Guo Y., Xu T., Li N., Cheng Q., Qiao D., Zhang B., Zhao S., Huang Q., Lin Q. (2019). Supramolecular Structure and Pasting/Digestion Behaviors of Rice Starches Following Concurrent Microwave and Heat Moisture Treatment. Int. J. Biol. Macromol..

[24] Li J., Zhou Q., Lin Q., Yang Q., Xiao H., Yang Y., Hang W. (2022). Structure and Physicochemical Properties of Indica Rice Starch with Different Degrees of Gelatinization. J. Chin. Cereals Oils Assoc..

[25] Liu K., Liu Q. (2020). Enzymatic Determination of Total Starch and Degree of Starch Gelatinization in Various Products. Food Hydrocoll..

[26] Zhang B., Li X., Liu J., Xie F., Chen L. (2013). Supramolecular Structure of A- and B-Type Granules of Wheat Starch. Food Hydrocoll..

[27] Li L., Chen J., Bai D., Xu M., Cao W., Ren G., Ren A., Duan X. (2022). Physicochemical, Pasting Properties and In Vitro Starch Digestion of Chinese Yam Flours as Affected by Microwave Freeze-Drying. Foods.

[28] Li N., Cai Z., Guo Y., Xu T., Qiao D., Zhang B., Zhao S., Huang Q., Niu M., Jia C. (2019). Hierarchical Structure and Slowly Digestible Features of Rice Starch Following Microwave Cooking with Storage. Food Chem..

[29] Wang S., Li C., Copeland L., Niu Q., Wang S. (2015). Starch Retrogradation: A Comprehensive Review. Compr. Rev. Food Sci. Food Saf..

[30] Cheng Z., Li J., Qiao D., Wang L., Zhao S., Zhang B. (2022). Microwave Reheating Enriches Resistant Starch in Cold-Chain Cooked Rice: A View of Structural Alterations during Digestion. Int. J. Biol. Macromol..

[31] Zhu F., Bertoft E., Li G. (2016). Morphological, Thermal, and Rheological Properties of Starches from Maize Mutants Deficient in Starch Synthase III. J. Agric. Food Chem..

[32] Chen X., Liu Y., Xu Z., Zhang C., Liu X., Sui Z., Corke H. (2021). Microwave Irradiation Alters the Rheological Properties and Molecular Structure of Hull-Less Barley Starch. Food Hydrocoll..

[33] Kumar Y., Singh L., Sharanagat V.S., Patel A., Kumar K. (2020). Effect of Microwave Treatment (Low Power and Varying Time) on Potato Starch: Microstructure, Thermo-Functional, Pasting and Rheological Properties. Int. J. Biol. Macromol..

[34] Ding X.-L., Wang L.-J., Li T.-T., Wang F., Quan Z.-Y., Zhou M., Huo Z.-Y., Qian J.-Y. (2021). Pre-Gelatinisation of Rice Flour and Its Effect on the Properties of Gluten Free Rice Bread and Its Batter. Foods.

[35] Liu Y., Chen J., Luo S., Li C., Ye J., Liu C., Gilbert R.G. (2017). Physicochemical and Structural Properties of Pregelatinized Starch Prepared by Improved Extrusion Cooking Technology. Carbohydr. Polym..

[36] Li S., Ward R., Gao Q. (2011). Effect of Heat-Moisture Treatment on the Formation and Physicochemical Properties of Resistant Starch from Mung Bean (Phaseolus Radiatus) Starch. Food Hydrocoll..

